# Development of an Agrobacterium-Mediated Stable Transformation Method for the Sensitive Plant *Mimosa pudica*

**DOI:** 10.1371/journal.pone.0088611

**Published:** 2014-02-12

**Authors:** Hiroaki Mano, Tomomi Fujii, Naomi Sumikawa, Yuji Hiwatashi, Mitsuyasu Hasebe

**Affiliations:** 1 Division of Evolutionary Biology, National Institute for Basic Biology, Okazaki, Japan; 2 School of Life Science, Graduate University for Advanced Studies, Okazaki, Japan; Virginia Tech, United States of America

## Abstract

The sensitive plant *Mimosa pudica* has long attracted the interest of researchers due to its spectacular leaf movements in response to touch or other external stimuli. Although various aspects of this seismonastic movement have been elucidated by histological, physiological, biochemical, and behavioral approaches, the lack of reverse genetic tools has hampered the investigation of molecular mechanisms involved in these processes. To overcome this obstacle, we developed an efficient genetic transformation method for *M. pudica* mediated by *Agrobacterium tumefaciens* (Agrobacterium). We found that the cotyledonary node explant is suitable for Agrobacterium-mediated transformation because of its high frequency of shoot formation, which was most efficiently induced on medium containing 0.5 µg/ml of a synthetic cytokinin, 6-benzylaminopurine (BAP). Transformation efficiency of cotyledonary node cells was improved from almost 0 to 30.8 positive signals arising from the intron-sGFP reporter gene by using Agrobacterium carrying a super-binary vector pSB111 and stabilizing the pH of the co-cultivation medium with 2-(*N*-morpholino)ethanesulfonic acid (MES) buffer. Furthermore, treatment of the explants with the detergent Silwet L-77 prior to co-cultivation led to a two-fold increase in the number of transformed shoot buds. Rooting of the regenerated shoots was efficiently induced by cultivation on irrigated vermiculite. The entire procedure for generating transgenic plants achieved a transformation frequency of 18.8%, which is comparable to frequencies obtained for other recalcitrant legumes, such as soybean (*Glycine max*) and pea (*Pisum sativum*). The transgene was stably integrated into the host genome and was inherited across generations, without affecting the seismonastic or nyctinastic movements of the plants. This transformation method thus provides an effective genetic tool for studying genes involved in *M. pudica* movements.

## Introduction

Being fixed in the soil, rooted plants have evolved a variety of strategies to survive stressful environments. Despite lacking muscular and nervous systems, which play pivotal roles in animal motility, certain plant species have acquired the ability to undergo rapid leaf movements in response to external stimuli [1]. The compound leaves of the leguminous species *Mimosa pudica* exhibit seismonastic movement within seconds [2] of being touched or subjected to other types of stimulation [3]. This rapid movement has been suggested to reduce predation risks [4] by scaring away predators [1], decreasing the visibility of the leaves [1], or exposing protective thorns that are usually obscured behind the leaves [5]. The physiological mechanisms underlying seismonastic movement have been studied extensively since the 19th century [6]. This movement is caused by a loss of turgor pressure in one half of the pulvinus (extensor [7]), which is located at the base of each primary petiole, pinna, and pinnule (leaflet) [6]. Nuclear magnetic resonance (NMR) imaging demonstrated that water is translocated from the extensor half to the other half (flexor [7]) of the pulvinus during the movement [8]. At the cellular level, individual “motor cells” in the extensor half of the pulvinus shrink following outflow of intracellular water [9], [10], which is accompanied by a large efflux of K^+^ and Cl^-^ ions [11], [12], [13], [14]. These rapid movements of water and ions are difficult to explain by a simple diffusion model [12], [15], suggesting that special mechanisms, such as solute-water co-transporters or contractile proteins, are involved in this process [15]. Pharmacological and cytological studies indicate that fragmentation of the actin cytoskeleton [16], [17], dephosphorylation of its tyrosine residues [17], [18], and changes in Ca^2+^ level [10], [19] in pulvinar motor cells participate in the movement. The seismonastic reaction can be propagated over a distance by an electrical action potential [20], which is likely transmitted through the protoxylem [20], [21] and the phloem [22]. Chemical substance(s) also contribute to the long-range transmission of the movement [23] and several candidate substances were identified by chemical analysis and bioassays [24], [25]. Mechanoreceptor cells in *M. pudica* have long been enigmatic; however, a recent study identified such cells on the tertiary pulvinus [26].

Despite many advances in our understanding of the physiology of seismonastic movements in *M. pudica*, the genetic mechanisms underlying this phenomenon remain to be unraveled, due to the lack of reverse genetic tools for this species. Until now, there was no technique for introducing desired genes into this plant's genome. Agrobacterium-mediated genetic transformation is widely used to generate transgenic plants [27] and is a well-established technique in model legumes such as *Medicago truncatula* [28] and *Lotus japonicus* [29]. However, transformation of other “recalcitrant” legumes, including *M. pudica*, remains challenging, because of the low frequency of shoot formation *in vitro* and the difficulty in transferring genes to cells that are capable of forming shoots [30], [31], [32].

In the present study, we developed an efficient Agrobacterium-mediated transformation method in *M. pudica*. To overcome the obstacles described above, we examined the shoot formation frequency of several types of explants and selected the cotyledonary node explant, which formed shoots at the highest frequency among the explants tested, as the starting material. We found that a super-binary vector, pSB111 [33], which exhibits improved transformation efficiencies due to the presence of additional virulence genes in the vector backbone [34], increases the number of transformed cells on the cotyledonary node. Furthermore, we demonstrated that controlling the pH during co-cultivation is required for efficient transformation. We thus established an effective transformation method for *M. pudica* that can be used to conduct reverse-genetic studies on the seismonastic movements of this plant.

## Materials and Methods

### Construction of T-DNA vectors and preparation of Agrobacterium cells

A DNA fragment containing the coding sequence of synthetic green fluorescent protein (*sGFP*) [35] was PCR amplified from pUGW4 [36] using a pair of primers (5′-AAAGT CGACT CGTGA GCAAG GGCGA GGAG-3′ and 5′-TTGAG CTCTT ACTTG TACAG CTCGT CCATG C-3′) and subcloned into the pCR-Blunt II-TOPO vector (Life Technologies, Carlsbad, USA). A DNA fragment containing the first intron of the castor bean *CAT-1* gene [37] was amplified from pIG121-Hm [38] with primers (5′-CTAAG CTTCG CAAGA CCCTT CCTC-3′ and 5′-ATTTC ACGGG TTGGG GTTTC TACAG GACG T-3′), digested with *Sal*I and *Xba*I, and inserted into the *Sal*I/*Xba*I site of the pCR-Blunt II-sGFP construct. Then a DNA fragment containing the intron-sGFP region was excised by digestion with *Sac*I and *Xba*I, and inserted into the *Sac*I/*Xba*I site of pIG121-Hm to produce the pIF121-Hm vector, in which the coding sequence of the beta-glucuronidase gene (*uidA*) [39] was replaced by that of *sGFP*. pIF121-Hm was then introduced into four different *Agrobacterium tumefaciens* (Agrobacterium) strains (AGL1, GV2260, EHA101, and LBA4404 [40]) by electroporation. A super-binary vector, pSB111-GFP, was prepared according to the method of Komari *et al.* [33], with modifications described below. A DNA fragment spanning the intron-*sGFP* sequence and the hygromycin phosphotransferase gene (*hpt*) was amplified from pIF121-Hm with primers (5′-GCAAC GCAAT TAATG TGAGT TAGCT C-3′ and 5′-GGGCT CGAGA GGGAA GAAAG CGAAA GGAG-3′), digested with *Hin*dIII and *Xho*I, and inserted into the *Hin*dIII/*Xho*I site of an intermediate vector, pSB11. The resultant construct, pSB11-GFP, was introduced into LBA4404 harboring pSB1 by electroporation and the pSB111-GFP vector was then produced by homologous recombination between pSB1 and pSB11-GFP in Agrobacterium. Agrobacterium cells harboring pIF121-Hm or pSB111-GFP were selected on LB medium containing 50 µg/ml hygromycin B (Life Technologies) and stored as glycerol stocks at −80°C.

### Preparation of cultivation media

Germination medium (GM) consisted of half-strength basal MS salts (1/2 MS; Wako, Osaka, Japan) and 0.2% (w/v) gellan gum (Phytagel; Sigma-Aldrich, St. Louis, USA) at pH 5.8. Shoot induction medium (SIM) consisted of 1/2 MS, 2% (w/v) sucrose, 1× Gamborg's vitamins (Sigma-Aldrich), 0.5 µg/ml 6-benzylaminopurine (BAP; Sigma-Aldrich), and 0.3% gellan gum at pH 5.8. Selection medium (SEM) was prepared by supplementing SIM with 15 µg/ml hygromycin B (Sigma-Aldrich) and 150 µg/ml cefotaxime sodium salt (Sanofi K.K., Tokyo, Japan). Co-cultivation medium (COM) consisted of 1/2 MS, 2% sucrose, 1× Gamborg's vitamins, 0.5 µg/ml BAP, and 0.1% (w/v) 2-(*N*-morpholino)ethanesulfonic acid (MES; Dojindo Laboratories, Kamimashiki-gun, Japan) at pH 6.1 or other values as indicated in the text. Each cultivation medium was prepared as follows: MS basal salts, sucrose, vitamins, BAP, 1-naphthaleneacetic acid (NAA; Sigma-Aldrich), and MES were dissolved in water and the pH was adjusted with KOH or HCl. Then the medium was combined with gellan gum and sterilized by autoclaving at 120°C for 20 min, or alternatively, by filtration through a 0.22-μm PES PLUS membrane (Asahi Glass, Tokyo, Japan) or 0.45-μm PVDF membrane (Millex HV; Merck-Millipore, Billerica, USA). Hygromycin B, cefotaxime, D-glucose, and acetosyringone were added after autoclaving. Cultivation media with minor modifications, for example those with different concentrations of phytohormones, were prepared in a similar manner.

### Sterilization of seeds

*M. pudica* “WASE (an early flowering accession)” seeds were purchased from Sakata Seed (Yokohama, Japan). Approximately 400 seeds in a 50-ml conical tube were washed briefly in 20 ml of 70% ethanol, and put under vacuum (-0.8 MPa) for 10 min in another 20 ml of 70% ethanol. Then the seeds were transferred to 20 ml of 50% commercial bleach (TOPVALU Kitchen Bleach; Aeon, Chiba, Japan) containing NaClO, NaOH, and alkylamineoxide, at concentrations not disclosed by the company, put under vacuum for 10 min, and then washed in another 20 ml of 50% bleach for 30 min with reciprocal shaking at 120 rpm. The seeds were rinsed with sterilized hot water (60°C) at least five times and soaked in 18 ml of hot water (60°C) for 10 min to remove seed coat waxes. After the addition of 2 ml of Plant Preservative Mixture (PPM; Plant Cell Technology, Washington DC, USA), the seeds were put under vacuum for 10 min and then shaken reciprocally at 120 rpm for 30 min. The seeds were placed in a 6×6 array in a Plant Box (a plastic cultivation box with dimensions of 60×60×100 mm; Asahi Glass, Tokyo, Japan) containing 80 ml of GM, and germinated at 25°C for 54 to 60 hours in the dark. Seedlings with hypocotyls of 3 to 8 mm in length were used for subsequent experiments.

### Preparation of explants and optimization of shoot induction conditions

**Figure 1:**
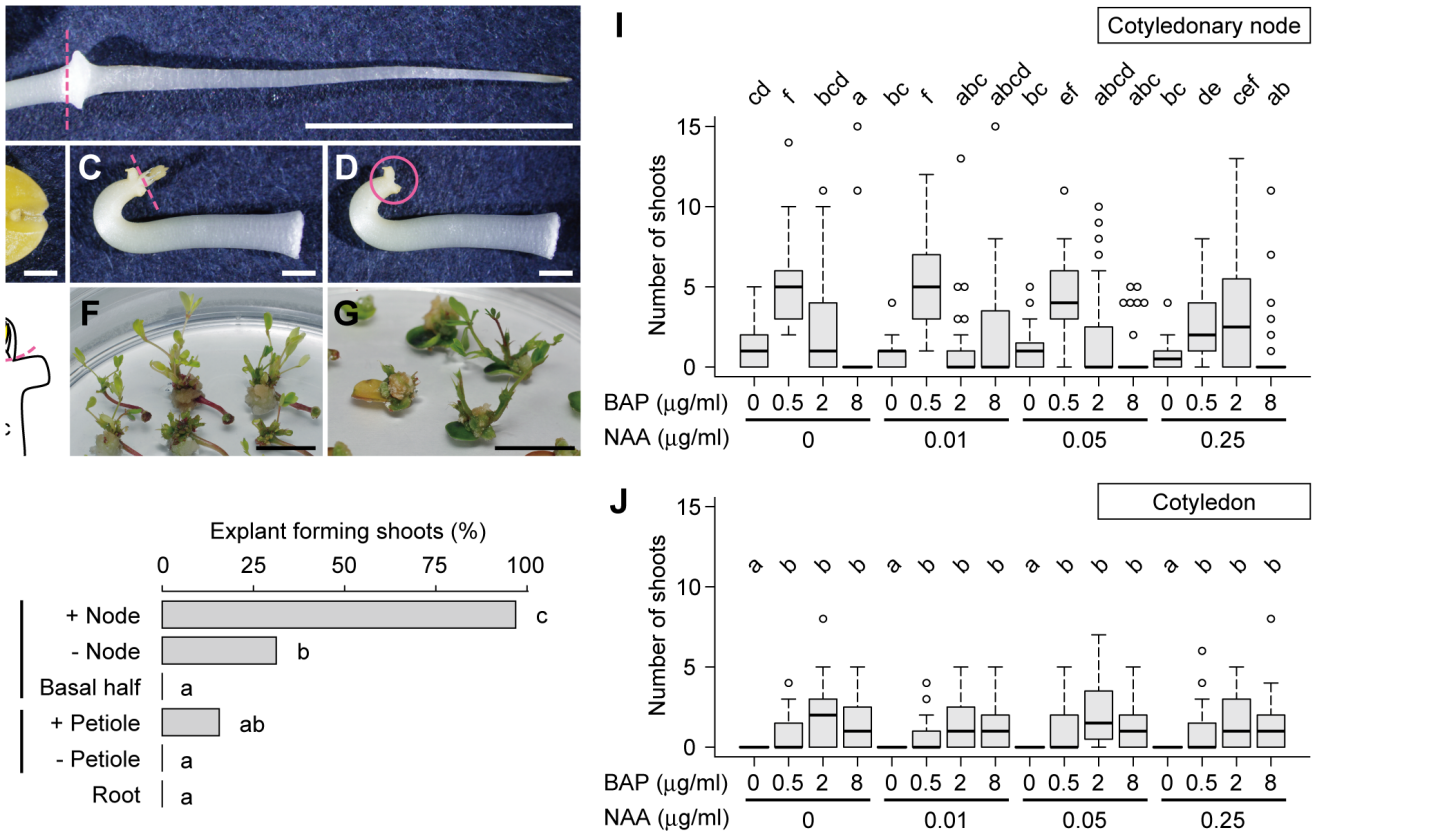
Shoot formation from *M. pudica* explants. A–E. Preparation of explants. A 2-day-old seedling cultured in the dark (A) was divided into the root, the cotyledons with petiole (B), and the remaining part (C). The epicotyl containing the shoot apex was then removed from the remaining part (C) to prepare the cotyledonary node explant (D) as illustrated in (E). Dashed lines in (A), (C), and (E) indicate the cutting positions. The circle in (D) indicates the position of the cotyledonary node. SA, shoot apex; Hc, hypocotyl. F, G. Shoot formation from the cotyledonary node (F) and petiolate cotyledon (G) explants after 4 and 6 weeks of cultivation in the presence of 0.5 µg/ml BAP, respectively. H. Comparison of the frequency of explants forming shoots after 4 weeks of cultivation with 0.5 µg/ml of BAP (n = 32). I, J. Effects of BAP and NAA on shoot formation from cotyledonary node (I) and petiolate cotyledon (J) explants after 4 and 6 weeks of cultivation, respectively. The distribution of the number of shoots formed per explant is shown as box-and-whisker plots (n = 32). Lower and upper whiskers indicate the range of values within 1.5 times the interquartile range from the box and circles indicate outliers. Significant differences were observed between two groups that do not share the same lowercase letter [P<0.05 by Fisher's exact test with Holm's P-value adjustment (H) or Steel-Dwass test (I, J)]. Scale bars, 1 cm (A, F, G), 1 mm (B–D).

Explants were prepared under a dissecting microscope in a laminar flow cabinet. Seedlings were dissected on three sheets of filter paper wetted with COM in a petri dish. After the seed coat was removed with forceps, the primary root and cotyledons were separated from the remaining part of the seedling using a surgical blade (No. 11; Feather, Osaka, Japan) (Figure 1). The epicotyl was cut off from the remaining part to produce the cotyledonary node explant and the associated hypocotyl. Sixteen explants were placed on SIM (25 ml in a 90×20 mm dish) or its derivatives containing different phytohormone concentrations. The explants were cultured at 25°C under 12-hour light (12L; with a light intensity of 120–180 µmol m^−2^ s^−1^)/12-hour dark (12D) cycles, and the medium was changed every 2 weeks. The number of shoots equal to or longer than 2 mm was counted on each explant after 4 or 6 weeks of cultivation.

### Transformation

An aliquot of the Agrobacterium stocks was streaked on solid LB medium containing 50 µg/ml hygromycin B and cultured at 30°C for 48 to 60 hours. A single colony was inoculated into 5 ml of liquid LB medium containing 25 µg/ml hygromycin B and pre-cultured at 28°C for 24 hours with rotatory shaking at 180 rpm. After large aggregates were removed by gravity settling, the liquid Agrobacterium culture was again inoculated into 40 ml of fresh liquid LB medium containing 25 µg/ml hygromycin B in a 200 ml baffled flask at a concentration of OD_600_ = 0.15. Then the culture was incubated at 28°C with rotatory shaking at 180 rpm for around 4 hours, until OD_600_ reached 0.6. The Agrobacterium cells were harvested by centrifugation at 5,000×g for 10 min at 25°C, resuspended in 20 ml of COM, centrifuged again, and resuspended in COM at a concentration of OD_600_ = 0.3. Finally, the Agrobacterium suspension was supplemented with 40 µg/ml acetosyringone, 0.2% (w/v) D-glucose, and, in some cases, 0.03% (v/v) Silwet L-77.

Twenty of the cotyledonary node explants were soaked in 10 ml of the Agrobacterium suspension with (or without) 0.03% Silwet L-77 in a glass test tube (16.5×165 mm). In some cases, the explants were sonicated with a Branson Sonifier 150 (Branson Ultrasonics, Danbury, USA) with three pulses of 5-s duration at the maximum output power (14 W). The explants were maintained under normal pressure or vacuum (−0.8 MPa) for 10 min and then collected with a tea strainer. The explants were transferred to a plastic dish (90×15 mm) containing 10 ml of the Agrobacterium suspension without Silwet L-77. Alternatively, the explants were directly transferred to the plastic dish without undergoing sonication, vacuum, or Silwet L-77 treatment. Then the dish was sealed with Parafilm (Bemis, Neenah, USA) and cultured for 3 days at 25°C in the dark. To monitor pH changes in co-cultivation medium, 200 µl of the medium was sampled at each time point and the pH was measured using a compact pH meter (Twin pH AS-212; As One, Osaka, Japan). After the co-cultivation, the explants were transferred to SEM (25 ml in a 90×15 mm dish) with forceps and continued to be cultured at 25°C under 12L (with a light intensity of 120–180 µmol m^−2^ s^−1^)/12D cycles, with the medium changed every 5 days. After 10 days of selection on SEM, GFP-positive signals located at the cotyledonary node region of each explant (Figure 1D) were counted visually under a fluorescence dissecting microscope SZX16 (Olympus, Tokyo, Japan) equipped with a SZX2-FGFPHQ filter. The number of GFP-positive signals was based on the number of spatially discrete spots, which were predominantly attributable to individual GFP-expressing cells but also included small clusters of cells. The number of explants possessing GFP-positive shoot buds was similarly counted after 30 days of selection. In this experiment, the shoot buds entirely consisting of GFP-positive cells were counted as GFP-positive buds, while chimeric buds containing only some GFP-positive cells were excluded.

Each explant was further cultured on SEM until a GFP-positive callus grew up to 2 mm in length. The GFP-positive callus was surgically excised from the explant and trimmed from GFP-negative tissue. The excised callus was cultured on SEM for an additional 5 days and then cultured on SIM (25 ml in a 90×20 mm dish), with the medium changed every 5 days. After the initiation of shoot elongation, the callus was transferred to a Plant Box containing 80 ml of SIM and continued to be cultured, with the medium changed every 10 days, until the shoots developed at least two compound leaves.

### Root induction and whole plant formation

Vermiculite (Fujimi Engei, Shizuoka, Japan) was poured into a Plant Box to a depth of approximately 3 cm, sterilized by autoclaving, and then irrigated with sterilized water, cultivation medium, or phytohormone solution. Cultivation media and water solidified with 0.3% and 1.5% Phytagel, respectively, were also prepared (80 ml per Plant Box). Different concentrations of Phytagel were used for cultivation media and water due to difficulties in solidifying media at low salt concentrations. Regenerated shoots of 2 to 3 cm in length and containing two or more compound leaves were cut with dissecting scissors and placed on vermiculite or gellan gum medium in a 3×3 array for each box. The shoots were kept at 25°C under 12L (with a light intensity of 120–180 µmol m^−2^ s^−1^)/12D cycles without changing the medium and the number of shoots forming any length of root was counted every 7 days. Twenty-seven shoots arising from three independent explants (n = 9 each) were examined for each experimental condition.

Once the root length of a regenerated plantlet reached 3 cm in total, the plantlet was transferred to a soft plastic pot (75×65 mm) containing an equal volume of granulated culture soil (Nippi Engei Baido 1; Nihon Hiryo, Tokyo, Japan) and vermiculite. It was cultured at 27°C under 14L (with a light intensity of 50–120 µmol m^−2^ s^−1^)/10D cycles for approximately 1 month and then transferred to a larger pot (120×100 mm). Liquid nutrient (Hyponex high grade; Hyponex Japan, Osaka, Japan) was occasionally supplied after the inflorescences became visible. Each inflorescence was enclosed in a small plastic bag 1 day before flowering and self-pollinated by rubbing it gently on the day of flowering. Collected seeds were stored at room temperature in a desiccator.

### Southern blot analysis

For genomic DNA extraction, immature leaves were sampled before leaflet opening. The leaves (100–200 mg in fresh weight) were frozen in liquid nitrogen and crushed to a fine powder with a mortar and pestle. The specimen was transferred to a 50 ml conical tube, combined with 20 ml of 2× CTAB buffer [2% (w/v) hexadecyltrimethylammonium bromide (CTAB), 1.4 M NaCl, 20 mM EDTA, 100 mM Tris-HCl (pH 8.0)] that had been heated to just below boiling point, and vortexed immediately. Then the sample was supplemented with 0.1% (v/v) 2-mercaptoethanol and incubated at 60°C for 1 hour with reciprocal shaking at 80 rpm. After the addition of 20 ml of chloroform, the sample was mixed on a rotator for 10 min and centrifuged at 10,000×g for 30 min at 25°C. The upper aqueous phase was transferred to a new tube, supplemented with 1/10 volume of 10% (w/v) CTAB containing 0.7 M NaCl, and re-extracted with chloroform. The sample solution was combined with an equal volume of 2-propanol and centrifuged at 10,000×g for 30 min at 25°C. The precipitation was rinsed with 5 ml of 70% ethanol, air-dried for 10 min, and resuspended in 400 µl of TE [10 mM Tris-HCl (pH 8.0), 1 mM EDTA] containing 0.1 mg/ml RNase A. The sample was incubated at 37°C for 1 hour with reciprocal shaking at 80 rpm, then supplemented with 1 mg/ml proteinase K, and further incubated at 56°C for 30 min with shaking. Genomic DNA in the extract was purified with Genomic-tip 100/G (Qiagen, Venlo, Netherlands) according to the manufacturer's instructions.

Genomic DNA fragments digested with *Eco*RI (5 µg per lane) were electrophoresed in 0.7% SeaKem GTG agarose (Takara Bio, Otsu, Japan) in 1× TAE buffer. Then the DNA fragments were transferred to a Hybond N+ membrane (GE Healthcare, Little Chalfont, UK) using the VacuGene XL Vacuum Blotting System (GE Healthcare). Southern hybridization was performed using an AlkPhos Direct Labeling and Detection System with CDP-Star (GE Healthcare). The DNA probe for *sGFP* was prepared by PCR with primers (5′-ATGGT GAGCA AGGGC GAGGA GC-3′ and 5′-TTACT TGTAC AGCTC GTCCA TGCC-3′) and the pSB111-GFP vector was used as template. Hybridization and subsequent primary washes were performed at 55°C and 65°C, respectively. Hybridization signals were detected using a LAS-3000 Mini luminescent image analyzer (Fujifilm, Tokyo, Japan).

## Results

### Optimization of shoot induction conditions

We examined shoot formation from several explants derived from 2-day-old *M. pudica* seedlings (Figure 1). After a 4-week cultivation in the presence of 0.5 µg/ml 6-benzylaminopurine (BAP), 97% (31 of 32) of the hypocotyls associated with the cotyledonary node (Figure 1D), hereafter referred to as cotyledonary node explants, formed shoots around the node (Figure 1F, H). Lower frequencies of shoot formation were observed from isolated cotyledons with petioles (5 of 32) and hypocotyls cut just beneath the node (10 of 32), and no shoot formation occurred from the basal halves of hypocotyls, roots, or cotyledons that lacked petioles (Figure 1H). These results indicate that tissues in and around the cotyledonary nodes of *M. pudica* have the ability to form shoots, as do those of other leguminous species [30], [31], [32].

We optimized the concentrations of two kinds of phytohormones, the cytokinin BAP and the auxin 1-naphthaleneacetic acid (NAA), both of which affect the number of shoots formed on the cotyledonary node in other leguminous species [41], [42], [43]. Shoots were most efficiently induced on the cotyledonary node in the presence of 0.5 µg/ml BAP and no NAA, which resulted in 5.2±0.5 (mean ± SE) shoots per explant after 4 weeks of cultivation (n = 32; Figure 1I). Shoot formation frequency of petiolate cotyledon explants was also examined at various phytohormone concentrations, but only 2.1±0.4 shoots per explant or fewer were induced, even after a longer cultivation period (n = 32; 6 weeks; Figure 1J). Based on these observations, the cotyledonary node was selected as the target tissue for Agrobacterium infection, and explants were cultured in medium supplemented with 0.5 µg/ml BAP in subsequent experiments.

### Agrobacterium-mediated transformation of cotyledonary nodes

**Figure 2:**
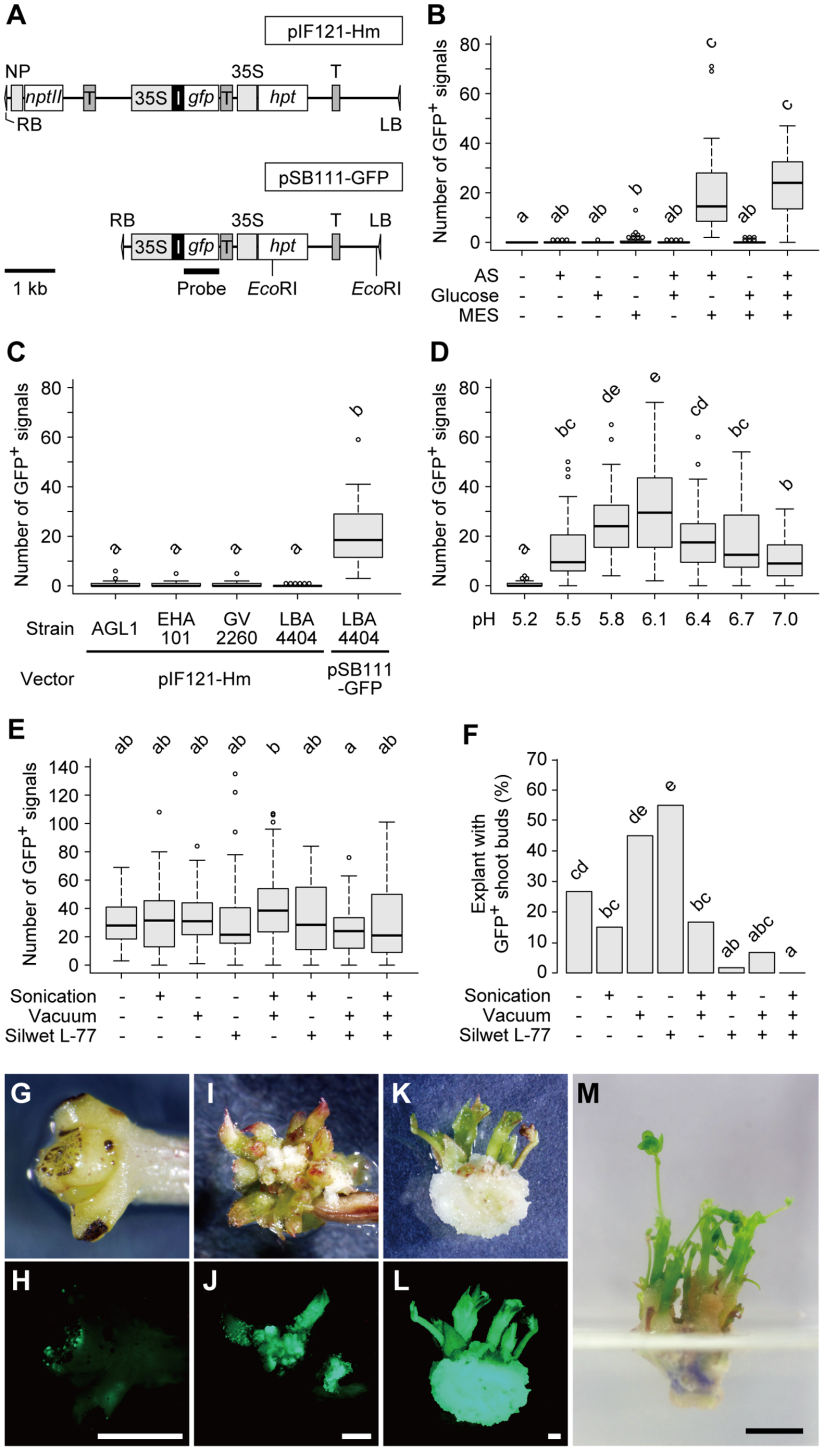
Optimization of Agrobacterium infection conditions. A. Schematic representations of the T-DNA regions of the binary vectors. The position of a probe used for Southern blot analysis and the *Eco*RI cutting sites are shown for pSB111-GFP. RB, right border [72]; LB, left border [72]; 35S, CaMV 35S promoter [73]; NP, *NOS* promoter [74]; T, *NOS* terminator [74]; I, first intron of the castor bean *CAT-1* gene [37]; *nptII*, *neomycin phosphotransferase II* gene [75]; *hpt*, *hygromycin phosphotransferase* gene [76]; and *gfp*, *sGFP* gene [35]. B. Effects of co-cultivation in the presence of acetosyringone (AS), D-glucose, and MES buffer on transformation efficiency (n = 40). The pH of each medium was adjusted to 5.8 before autoclaving. C. Effects of Agrobacterium strains and vectors on transformation efficiency (n = 40). Co-cultivation medium containing acetosyringone, glucose, and MES (pH 5.8) was used. D. Effects of initial pH of co-cultivation media (n = 60). E, F. Effects of sonication, vacuum infiltration, and Silwet L-77 detergent treatments prior to co-cultivation (n = 60). The number of GFP-positive signals on the cotyledonary node of each explant was counted after 10 days of selection (B–E). The frequency of explants possessing GFP-positive shoot buds was counted after 30 days of selection (F). Significant differences were observed between two groups that do not share the same lowercase letter [P<0.05 by the Steel-Dwass test (B–E) or Fisher's exact test with Holm's P-value adjustment (F)]. G–J. Bright-field (G, I) or green fluorescent (H, J) images of cotyledonary node explants after 10 (G, H) or 30 (I, J) days of selection. K, L. Bright-field (K) or green fluorescent (L) images of an isolated GFP-positive callus after 51 days of selection. M. After an additional 21 days of cultivation (a total of 72 days), the same callus shown in (K) formed multiple shoots with compound leaves. Scale bars, 1 mm (G–L), 1 cm (M).

For the Agrobacterium-mediated transformation of cotyledonary node cells, we prepared two kinds of binary vectors: a conventional binary vector, pIF121-Hm, and a super-binary vector, pSB111-GFP, which possesses additional virulence genes [33], [34]. The T-DNA region of each vector carries an intron-sGFP reporter gene (Figure 2A), which can be used to selectively visualize transformed cells in living plant tissues, but does not label Agrobacterium cells [37].

*Agrobacterium tumefaciens* infection is triggered by the transcriptional activation of its virulence genes [44] in response to phenolic compounds such as acetosyringone [45], monosaccharides [46], [47], and acidic pH values [48]. We thus examined the effects of supplementing the co-cultivation medium with acetosyringone, D-glucose, and MES buffer adjusted to pH 5.8, alone or in combination, on transformation efficiency (Figure 2B). The number of GFP-positive signals in the cotyledonary node increased in the presence of both acetosyringone and MES (Figure 2B). Although the addition of glucose further increased the transformation efficiency of explants treated with acetosyringone and MES buffer, the increase was not significant (Figure 2B). However, since the addition of glucose did potentially increase the transformation efficiency, we used all three compounds in the subsequent experiments. A comparison of the two binary vectors and Agrobacterium strains demonstrated that the transformation efficiency was higher when pSB111-GFP was combined with Agrobacterium strain LBA4404 than when pIF121-Hm was combined with any of four different Agrobacterium strains (Figure 2C). These results suggest that the addition of acetosyringone and of a buffer capable of maintaining an acidic pH enhance the transformation efficiency of *M. pudica*, as does the use of a super-binary vector.

**Figure 3:**
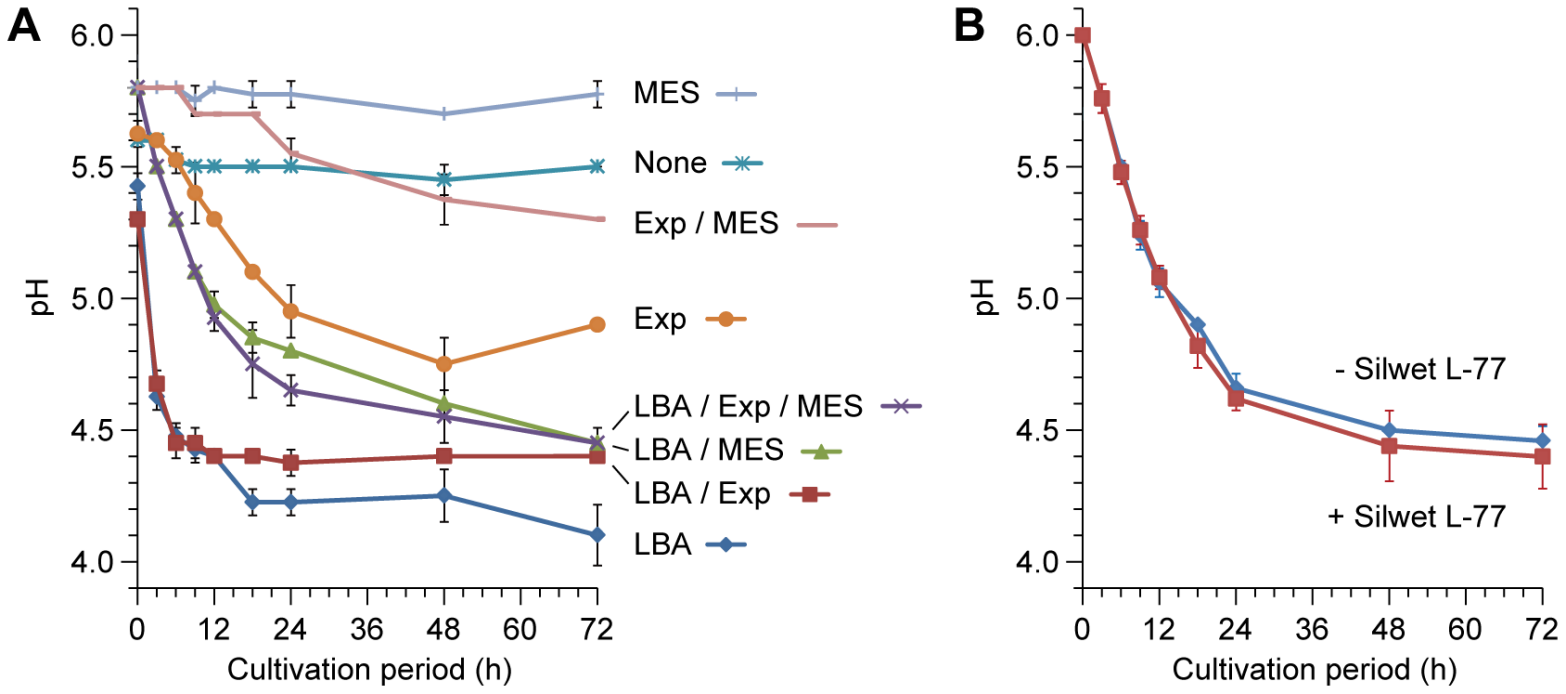
Changes in medium pH during co-cultivation. A. Effects of Agrobacterium strain LBA4404 harboring pSB111-GFP (LBA), cotyledonary node explants (Exp), and MES buffer (MES) on medium pH (n = 4). The co-cultivation media were initially adjusted to pH 5.8 and sterilized by filtration to circumvent the pH decrease caused by autoclaving. In the absence of MES buffer, pH values had already declined in the time it took to prepare the Agrobacterium suspension in co-cultivation medium (∼30 minutes). B. Changes in pH of MES-buffered medium initially adjusted to pH 6.1 (n = 5). The medium was sterilized by autoclaving and then used for co-cultivation of Agrobacterium and explants. The effect of Silwet L-77 treatment prior to co-cultivation was also examined. Data are the means ± SD. The pH of the medium was measured at 0, 3, 6, 9, 12, 18, 24, 48, and 72 hours after the initiation of cultivation.

We further assessed the effect of pH on Agrobacterium infection. As reported previously [49], the addition of MES to cultivation media reduced the amount of pH changes during autoclaving (Figure S1A). However, the smaller change in pH after autoclaving was not the direct cause of the improved infection efficiency, because the cotyledonary nodes of explants cultured on filtration-sterilized, non-buffered medium had almost no positive signals, as did those cultured on autoclaved, non-buffered medium (Figure S1B). Monitoring the pH of the medium during the co-cultivation period revealed that the pH in the non-buffered medium containing Agrobacterium and the explants dropped below 4.7 within the first three hours (Figure 3A). A similar decrease in pH was observed in the non-buffered medium containing only Agrobacterium, but not in the medium alone or in medium containing only explants (Figure 3A), suggesting that the conspicuous acidification of the co-cultivation medium was mainly caused by Agrobacterium. The addition of MES buffer relieved, but did not completely prevent, the excessive acidification and kept the pH of the medium above 5.0 for at least 9 hours during co-cultivation (Figure 3A). The addition of MES also improved the transformation efficiency when using a solid co-cultivation medium, but to a lesser extent than the liquid co-cultivation medium (Figure S1C). Optimization of the initial pH value demonstrated that transformation was most efficient in liquid co-cultivation medium adjusted to pH 6.1 (Figure 2D). This value was higher than those reported for the maximum induction of virulence genes in octopine-type Agrobacterium strains (pH 5.2 to 5.3) [48], [50], and possibly counterbalanced the pH decrease during co-cultivation (Figure 3B).

To further improve the transformation efficiency, we examined the effects of sonication [51] and vacuum infiltration [52] prior to the co-cultivation period. We also gauged the effects of transiently adding a detergent, Silwet L-77 [53], [54], to the Agrobacterium suspension at 0.03% (v/v) during the sonication and/or vacuum treatments. Compared to the control experiment, none of the treatments, individually or combined, significantly altered the number of GFP-positive signals after 10 days of selection (Figure 2E). On the other hand, the number of explants forming GFP-positive shoot buds after 30 days varied depending on the treatments (Figure 2F). A significant, two-fold increase was observed in the explants treated only with Silwet L-77 (Figure 2F), suggesting that the detergent facilitates Agrobacterium infection of cells that are capable of forming shoots, which are possibly situated deep inside the cotyledonary node. The additional use of sonication and/or vacuum in combination with the Silwet L-77 treatment reduced the emergence of GFP-positive shoot buds (Figure 2F), possibly due to the increased damage of cells at the cotyledonary node.

Taken together, the transformation efficiency of the cotyledonary node of *M. pudica* was drastically improved by three different factors: the use of the super-binary vector, the addition of MES buffer to the co-cultivation medium, and transient treatment with Silwet L-77 before co-cultivation.

### Root induction and whole plant formation

**Figure 4:**
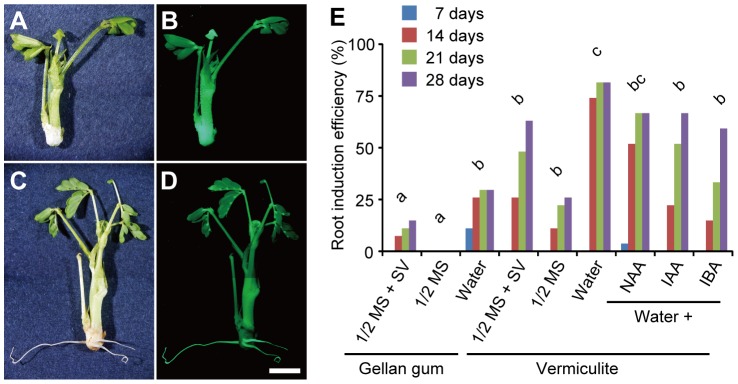
Optimization of root induction conditions. A–D. Bright-field (A, C) and green fluorescent (B, D) images of a transformed shoot before (A, B) and after (C, D) 14 days of cultivation in irrigated vermiculite. Scale bar, 5 mm. E. Comparison of root induction conditions (n = 27). A statistical analysis was conducted using the frequencies at 14 days of cultivation. Significant differences (P<0.05 by Fisher's exact test with Holm's P-value adjustment) were observed between two groups that do not share the same lowercase letter. 1/2 MS, half-strength MS salts; SV, 2% sucrose and 1× Gamborg's vitamins.

After 1 month or longer of selection with hygromycin B, transformed cells in the cotyledonary node formed GFP-positive calluses with shoot buds (Figure 2I–J). These calluses were surgically isolated from the explants and continued to be cultured on SIM for further shoot development (Figure 2K–M). Well-developed shoots possessing at least two compound leaves (Figure 4A, B) were used in a root induction experiment in which three nutrient conditions (water, 1/2 MS, or 1/2 MS containing sucrose and vitamins) and two supporting materials (gellan gum or vermiculite) were tested. For both supporting materials, higher root induction efficiencies were obtained with water than with the MS-based media (Figure 4E), suggesting that poor nutrient conditions favored rooting. Vermiculite increased root induction efficiencies to a greater extent than did gellan gum (Figure 4E), possibly due to the improved permeability to air [55]. Roots were most efficiently induced by vermiculite supplied with water, which resulted in rooting of 81% (22 of 27) of the regenerated shoots after 21 days of cultivation (Figure 4E). This efficiency, together with the fact that the transformed shoots can readily be multiplied by vegetative propagation on SIM, ensures the root induction on practically all transformed shoots. We also examined the effects of three auxins, NAA, indole-3-acetic acid (IAA), and indole-3-butyric acid (IBA), all of which were used for root induction in various plants [43], [56], [57], [58]. None of these compounds, however, improved the root induction efficiency of our system any further, when used at a concentration of 0.5 µg/ml (Figure 4E). The resultant plantlets were transferred to soil after their roots reached 3 cm in total length (Figure 4C, D) and their establishment in the soil was confirmed by further cultivation.

**Figure 5:**
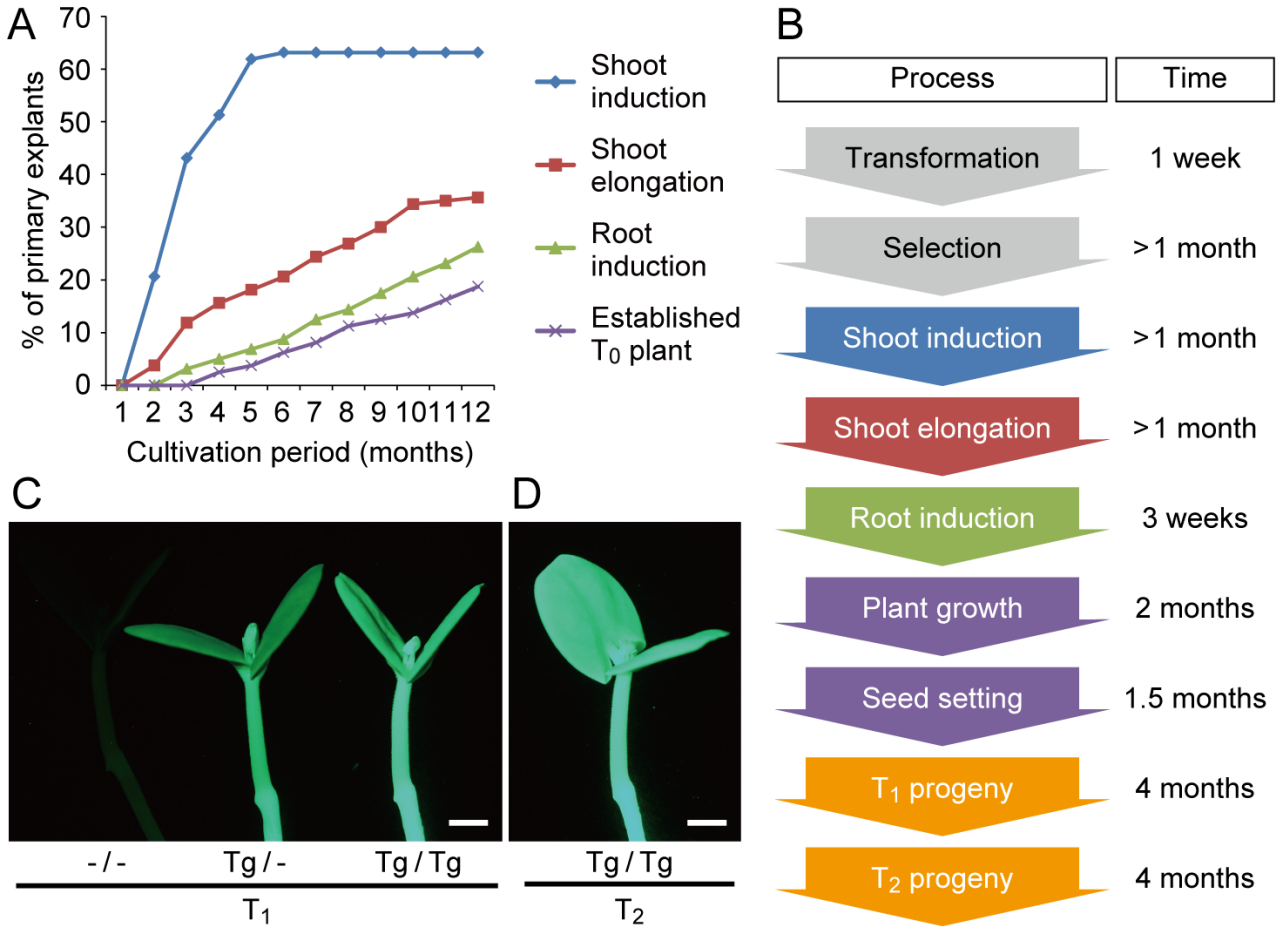
Establishment of transgenic plants. A. Time course of establishment of transgenic T_0_ plants from cotyledonary node explants (n = 160). Each line indicates the frequency of Agrobacterium-infected explants that reached the process indicated in (B). B. Schematic representation of the entire transformation procedure. C. Green fluorescent images of T_1_ seedlings of a single T-DNA insertion line (#1 in Figure 6). The zygosity of T_1_ progeny [non-transformant (−/−), hemizygote (Tg/−) or homozygote (Tg/Tg)] produced by self-crossing of a T_0_ plant could be determined based on fluorescence intensity of the plantlet. D. Green fluorescent image of homozygous T_2_ seedling produced by self-crossing of a homozygous T_1_ plant. Scale bar, 2 mm.

Using the optimized conditions described above, we evaluated the transformation efficiency of *M. pudica* throughout the entire procedure. A total of 160 cotyledonary node explants were subjected to the Agrobacterium-mediated transformation, and monitored for 12 months after co-cultivation (Figure 5A). Sixty-three percent (101 of 160) of the explants formed GFP-positive calluses during selection and more than a half of them (57 of 101) initiated shoot elongation on SIM. Forty-two of the 57 shoots developed two or more compound leaves and 30 of these successfully rooted and became established in the soil. These results demonstrated that 18.8% of the explants (30 of 160) produced at least one independent line of T_0_ plants (Figure 5A). The number of transgenic T_0_ plants continued to increase even after 12 months of cultivation (Figure 5A), suggesting that the efficiency would further increase with time. On the other hand, four independent T_0_ plants (derived from 2.5% of the explants) became established in as little as 4 months (Figure 5A), enabling us to recover their T_1_ progeny within a total of 8 months (Figure 5B).

### Molecular and biological analyses of transgenic plants

**Figure 6:**
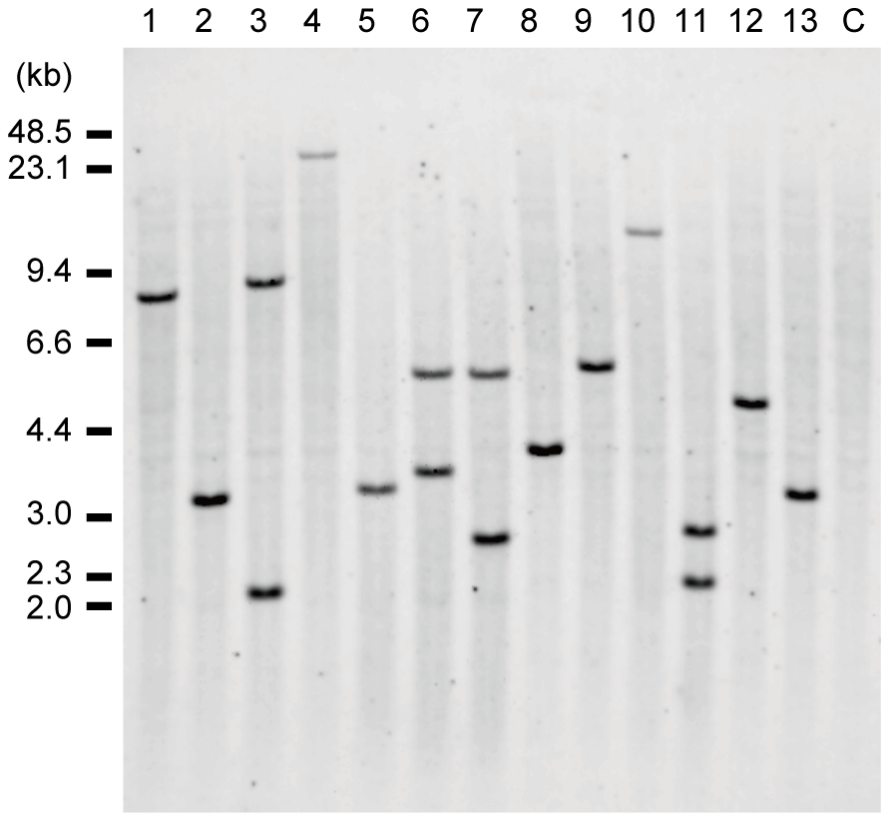
Genomic Southern blot analysis of T_0_ plants. Genomic DNAs of 13 independent T_0_ plants (lines #1-13) and a non-transformed plant (C) were analyzed by *Eco*RI digestion and detection of the *sGFP* sequence. The size of several bands is shorter than the minimal length expected from the intact T-DNA sequence (3.0 kb; Figure 2A), suggesting that the T-DNA sequence had undergone truncation and/or rearrangement.

We performed a genomic Southern blot analysis on regenerated T_0_ plants using the sGFP sequence as a probe (Figure 2A). Among 13 independent lines tested, approximately two-thirds of transgenic plants (9 of 13) possessed a single T-DNA insertion, while the others (4 of 13) had two insertions (Figure 6). This simple pattern of insertion represents an advantage of the present method over particle bombardment, which is used to transform other leguminous species [30], [31], but which often results in complex patterns of DNA insertions [59], [60].

Transmission of the transgene to T_1_ progeny was confirmed in all lines tested (n = 10) by observing the GFP fluorescence (Figure 5C). In most cases (9 of 10), the segregation ratio of the GFP fluorescence in a selfed T_1_ progeny was in good agreement with that expected from the number of T-DNA insertions (Table S1; 3∶1 and 15∶1 for one and two T-DNA insertions, respectively). These results provide further evidence for the simplicity of T-DNA insertion patterns produced by the present method and also indicate the non-chimeric nature of each T_0_ plant. Transmission of the transgene to T_2_ progeny was also confirmed for one line (Figure 5D), demonstrating the stable transmission of transgenes across generations.

Finally, the transgenic plants were examined for their ability to undergo characteristic movements. All of the T_0_ (n = 70) and T_1_ (n = 10) plants showed both seismonastic movement in response to touch (Video S1) and nyctinastic movement (data not shown), suggesting that the transformation procedure presented here does not impair these movements. In sum, the present study provides a genetic tool to investigate the molecular mechanisms underlying the intriguing movements of *M. pudica*.

## Discussion

In this study, we developed a robust protocol for the genetic transformation of *M. pudica*. A key improvement for the successful transformation of *M. pudica* was the use of MES buffer to maintain the pH during co-cultivation. Although the pH-dependent activation of Agrobacterium virulence genes was previously demonstrated [48], [50] and several studies emphasized the importance of buffering agents in co-cultivation media [61], [62], the requirement to stabilize the pH with buffering agents seems to depend on the transformation system being used. For example, only one-quarter (17 of 67) of the transformation methods given in a protocol book [27] that covers a wide range of plant species and transformation systems describes the use of buffer reagents during Agrobacterium preparation and/or co-cultivation. This variability may be due to differences in other conditions that possibly affected pH stability, such as the composition of co-cultivation media, the plant species and tissues being used, the Agrobacterium strains, and procedures used to prepare them. Although a previous study reported that MES reduces the transformation efficiency [63], the present study indicates that controlling the pH with buffering agents may improve the efficiency of Agrobacterium-mediated transformation. The direction of pH change also varied with the transformation system being used; the pH dropped to below 4.5 in a trumpet lily (*Lilium* x *formolongi*) system [62] and the present study, whereas it rose to 7.2 in a Tepary bean (*Phaseolus acutifolius*) system [61]. These observations, together with the finding that the optimal initial pH of the co-cultivation medium (pH 6.1) differed from that reported for the maximal activation of the virulence genes in other octopine-type strains (pH 5.2 to 5.3) [48], [50], indicate the importance of pH optimization for each transformation system.

The transformation efficiency of the present study (18.8%) is comparable to efficiencies obtained for extensively studied, recalcitrant legumes such as soybean (16.4%) [64] and pea (13.5%) [65]. This level of transformation efficiency is sufficient for conventional transgenic analyses that introduce a limited number of foreign DNAs of interest. On the other hand, further improvement of the method may be needed for high-throughput genetic screenings, such as insertional mutagenesis [66], activation tagging [67], and the FOX hunting system [68], which rely on a large number of transgenic plants. One possible approach for improvement would be to increase the Agrobacterium infection efficiency with thiol compounds, which are effective for the transformation of soybean [69], [70]. Another approach would be to increase the frequency of shoot formation from the transformed calluses, because only 30% (30 of 101) of the calluses produced well-developed shoots, even after a long cultivation period (Figure 5A). Further optimization of cultivation conditions, such as temperature, lighting, nutritional composition, and phytohormones, would increase the transformation efficiency and/or accelerate shoot formation.

In this study, the transgenic plants were recovered via a combination of hygromycin selection and fluorescence-based visual selection, in which GFP-positive transgenic calluses were surgically isolated from surrounding non-transgenic regions. Compared to hygromycin selection alone, this dual selection system facilitates and accelerates the establishment of transgenic T_0_ plants that consist entirely of transformed cells. On the other hand, our preliminary observation indicated that 70% (21 of 30) of the regenerated shoots (>5 mm) exhibited GFP fluorescence after 60 days of cultivation on SEM. This result suggests that transformants can also be recovered using antibiotic selection alone, although further investigation is required to evaluate the recovery rate of transgenic plants under these conditions.

Despite the recent development of new reverse genetic tools, such as virus-induced gene silencing (VIGS) [71], Agrobacterium-mediated transformation still plays a pivotal role in plant biology research. In the present study, we establish a method whereby this invaluable genetic technique may be applied to *M. pudica*, a classic model organism in plant physiology.

## Supporting Information

Figure S1 **Effects of MES buffer on transformation efficiency.** A. Changes in pH of co-cultivation media after autoclaving. Data are the means ± SD (n = 3). A diagonal line is shown for clear visualization of the pH changes from initial values. B. Comparison of sterilization methods of co-cultivation media in the presence or absence of 0.1% MES buffer (n = 20). Each co-cultivation medium was adjusted to pH 5.8 before sterilization and supplemented with both acetosyringone and glucose. C. Comparison of liquid and solid co-cultivation media in the presence or absence of 0.1% MES buffer (n = 20). The pH of each co-cultivation medium was adjusted to 5.8 before autoclaving. Gellan gum (0.3%) was used to solidify the solid co-cultivation media. The number of GFP-positive signals on the cotyledonary node of each explant was counted after 10 days of selection. Significant differences (P<0.05 by the Steel-Dwass test) were observed between two groups that do not share the same lowercase letter (B, C). D, E. Comparison of sterilization methods of co-cultivation medium optimized for transformation (n = 80). Co-cultivation medium containing acetosyringone, glucose, and MES buffer (pH 6.1) and the treatment with Silwet L-77 prior to co-cultivation were used in this experiment. No significant differences were observed in either the number of GFP-positive signals after 10 days of selection (D; by the Mann-Whitney *U*-test) or the frequency of explants possessing GFP-positive shoot buds after 30 days of selection (E; by Fisher's exact test).
(TIF)

Table S1 **Segregation of GFP expression in selfed T_1_ progeny.**
(TIF)

Video S1 **Movie of the seismonastic movement of transgenic** ***M. pudica*** **.** A homozygous T_1_ seedling (10 days old) is shown. Green and red signals represent the GFP fluorescence and the autofluorescence of chloroplasts, respectively. This movie was taken using a SZX16 microscope equipped with a SZX2-FGFP long-pass filter and coupled to a DP71 digital camera (Olympus).
(WMV)
