# Correction: Development of an Agrobacterium-Mediated Stable Transformation Method for the Sensitive Plant *Mimosa pudica*

**DOI:** 10.1371/journal.pone.0097211

**Published:** 2014-05-02

**Authors:** The PLOS ONE Staff

Figure 1 is incorrect. The publisher apologizes for this error. The authors have provided a correct version here.

**Figure 1:**
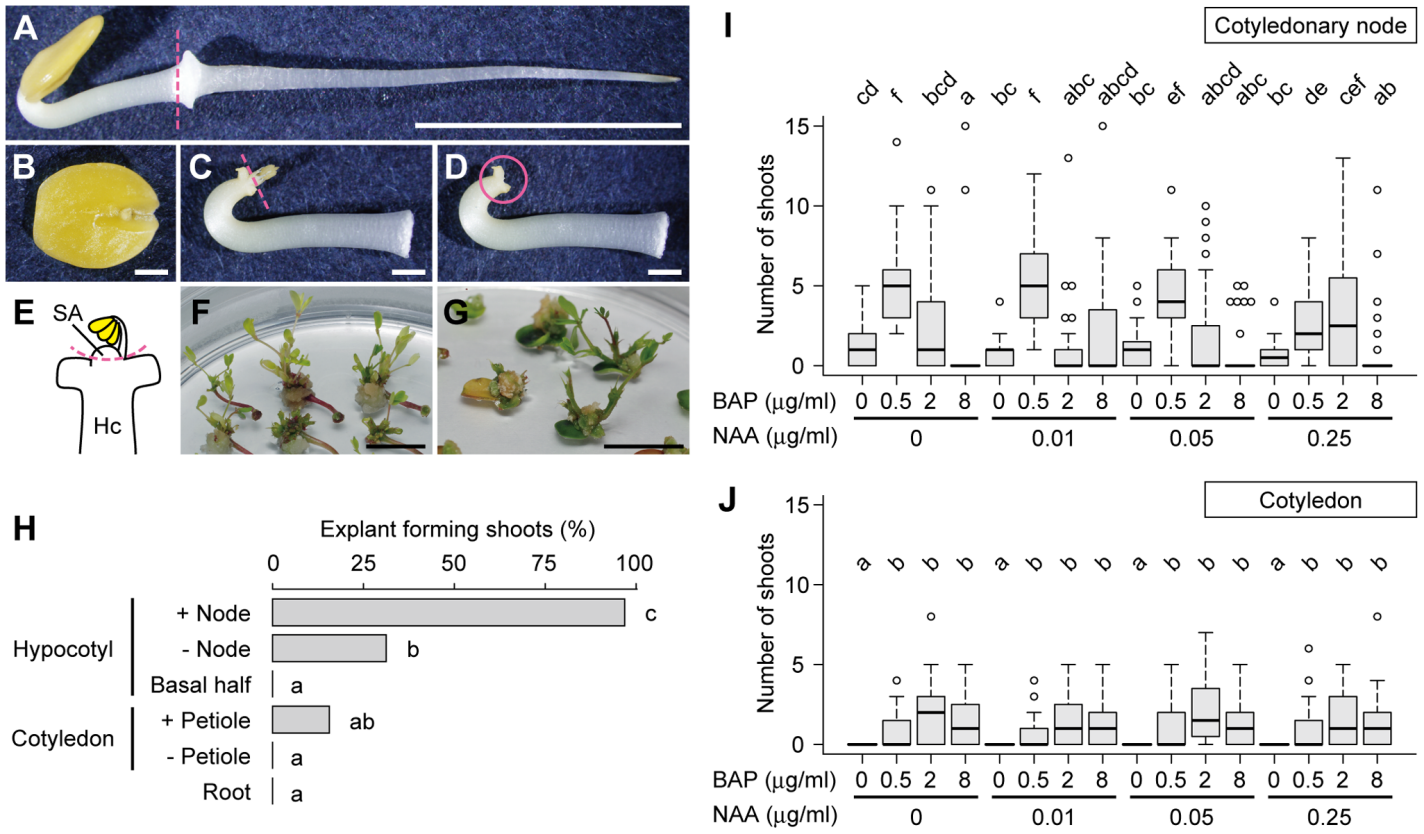
Shoot formation from *M. pudica* explants. A–E. Preparation of explants. A 2-day-old seedling cultured in the dark (A) was divided into the root, the cotyledons with petiole (B), and the remaining part (C). The epicotyl containing the shoot apex was then removed from the remaining part (C) to prepare the cotyledonary node explant (D) as illustrated in (E). Dashed lines in (A), (C), and (E) indicate the cutting positions. The circle in (D) indicates the position of the cotyledonary node. SA, shoot apex; Hc, hypocotyl. F, G. Shoot formation from the cotyledonary node (F) and petiolate cotyledon (G) explants after 4 and 6 weeks of cultivation in the presence of 0.5 µg/ml BAP, respectively. H. Comparison of the frequency of explants forming shoots after 4 weeks of cultivation with 0.5 µg/ml of BAP (n  =  32). I, J. Effects of BAP and NAA on shoot formation from cotyledonary node (I) and petiolate cotyledon (J) explants after 4 and 6 weeks of cultivation, respectively. The distribution of the number of shoots formed per explant is shown as box-and-whisker plots (n  =  32). Lower and upper whiskers indicate the range of values within 1.5 times the interquartile range from the box and circles indicate outliers. Significant differences were observed between two groups that do not share the same lowercase letter [P<0.05 by Fisher's exact test with Holm's P-value adjustment (H) or Steel-Dwass test (I, J)]. Scale bars, 1 cm (A, F, G), 1 mm (B–D).
