# Supplementary figures and images for: Development of an Agrobacterium-Mediated Stable Transformation Method for the Sensitive Plant Mimosa pudica
Source: PLoS One. 2014 Feb 12;9(2):e88611. doi: 10.1371/journal.pone.0088611 (PMC3922943; doi:10.1371/journal.pone.0088611)

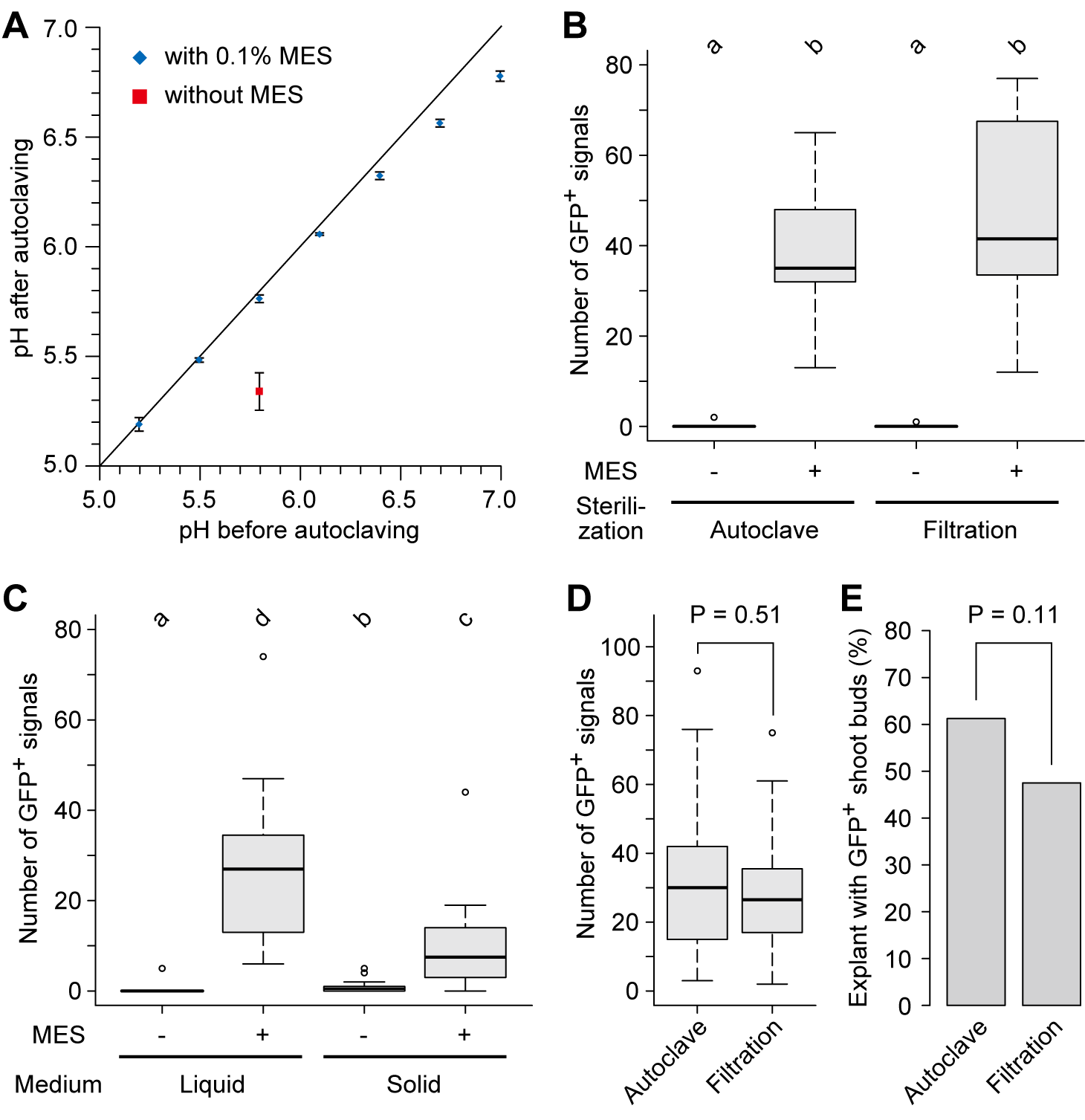

Supplement: Figure S1 — Effects of MES buffer on transformation efficiency. A. Changes in pH of co-cultivation media after autoclaving. Data are the means ± SD (n = 3). A diagonal line is shown for clear visualization of the pH changes from initial values. B. Comparison of sterilization methods of co-cultivation media in the presence or absence of 0.1% MES buffer (n = 20). Each co-cultivation medium was adjusted to pH 5.8 before sterilization and supplemented with both acetosyringone and glucose. C. Comparison of liquid and solid co-cultivation media in the presence or absence of 0.1% MES buffer (n = 20). The pH of each co-cultivation medium was adjusted to 5.8 before autoclaving. Gellan gum (0.3%) was used to solidify the solid co-cultivation media. The number of GFP-positive signals on the cotyledonary node of each explant was counted after 10 days of selection. Significant differences (P<0.05 by the Steel-Dwass test) were observed between two groups that do not share the same lowercase letter (B, C). D, E. Comparison of sterilization methods of co-cultivation medium optimized for transformation (n = 80). Co-cultivation medium containing acetosyringone, glucose, and MES buffer (pH 6.1) and the treatment with Silwet L-77 prior to co-cultivation were used in this experiment. No significant differences were observed in either the number of GFP-positive signals after 10 days of selection (D; by the Mann-Whitney U-test) or the frequency of explants possessing GFP-positive shoot buds after 30 days of selection (E; by Fisher's exact test). (TIF) [file pone.0088611.s001.tif]

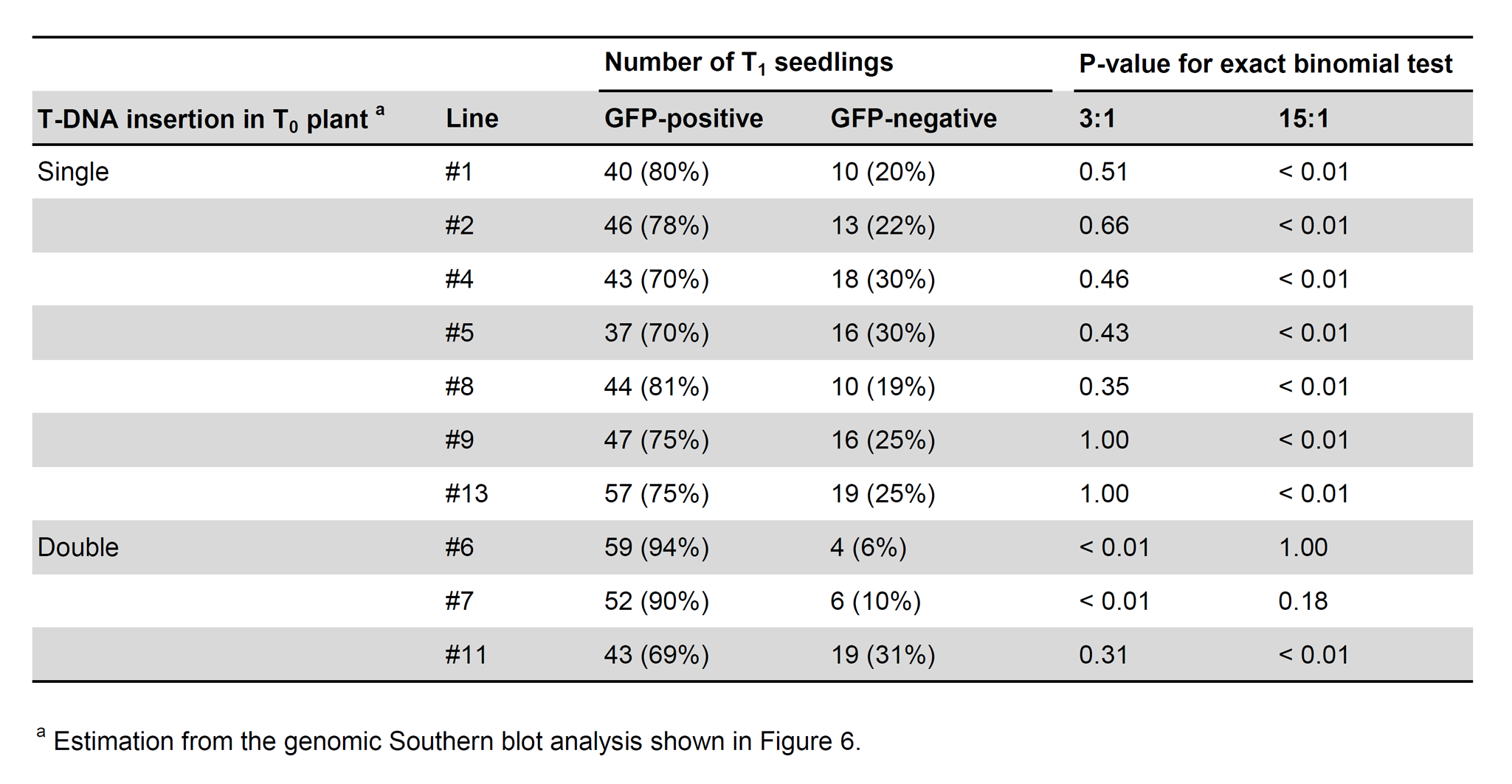

Supplement: Table S1 — Segregation of GFP expression in selfed T1 progeny. (TIF) [file pone.0088611.s002.tif]
